# *Arabidopsis* SUMO E3 Ligase SIZ1 Interacts with HDA6 and Negatively Regulates HDA6 Function during Flowering

**DOI:** 10.3390/cells10113001

**Published:** 2021-11-03

**Authors:** Sujuan Gao, Xueqin Zeng, Jianhao Wang, Yingchao Xu, Chunwei Yu, Yishui Huang, Feng Wang, Keqiang Wu, Songguang Yang

**Affiliations:** 1 Key Laboratory of Green Processing and Intelligent Manufacturing of Lingnan Specialty Food, College of Light Industry and Food Science, Zhongkai University of Agriculture and Engineering, Ministry of Agriculture, Guangzhou 510225, China; gaoshj@126.com; 2 Guangdong Provincial Key Laboratory of New Technology in Rice Breeding, Rice Research Institute, Guangdong Academy of Agricultural Sciences, Guangzhou 510640, China; sszxq@126.com (X.Z.); fwang1631@163.com (F.W.); 3 Innovative Center of Molecular Genetics and Evolution, School of Life Sciences, Guangzhou University, Guangzhou 510000, China; xn2112wjh@163.com; 4 Key Laboratory of South China Agricultural Plant Molecular Analysis and Genetic Improvement, Guangdong Provincial Key Laboratory of Applied Botany, South China Botanical Garden, Chinese Academy of Sciences, Guangzhou 510650, China; yingchaoxu2021@163.com (Y.X.); illvm@163.com (Y.H.); 5 Institute of Plant Biology, National Taiwan University, Taipei 106, Taiwan; wayneyu2@yahoo.com.tw; 6 Guangdong Key Laboratory for New Technology Research of Vegetables, Vegetable Research Institute, Guangdong Academy of Agricultural Sciences, Guangzhou 510640, China

**Keywords:** HDA 6, SUMO E3 ligase SIZ1, flowering, *Arabidopsis*

## Abstract

The changes in histone acetylation mediated by histone deacetylases (HDAC) play a crucial role in plant development and response to environmental changes. Mammalian HDACs are regulated by post-translational modifications (PTM), such as phosphorylation, acetylation, ubiquitination and small ubiquitin-like modifier (SUMO) modification (SUMOylation), which affect enzymatic activity and transcriptional repression. Whether PTMs of plant HDACs alter their functions are largely unknown. In this study, we demonstrated that the *Arabidopsis* SUMO E3 ligase SAP AND MIZ1 DOMAIN-CONTAINING LIGASE1 (SIZ1) interacts with HISTONE DEACETYLASE 6 (HDA6) both in vitro and in vivo. Biochemical analyses indicated that HDA6 is not modified by SUMO1. Overexpression of *HDA6* in *siz1-3* background results in a decreased level of histone H3 acetylation, indicating that the activity of HDA6 is increased in *siz1-3* plants. Chromatin immunoprecipitation (ChIP) assays showed that SIZ1 represses HDA6 binding to its target genes *FLOWERING LOCUS C* (*FLC*) and *MADS AFFECTING FLOWERING 4* (*MAF4*), resulting in the upregulation of *FLC* and *MAF4* by increasing the level of histone H3 acetylation. Together, these findings indicate that the *Arabidopsis* SUMO E3 ligase SIZ1 interacts with HDA6 and negatively regulates HDA6 function.

## 1. Introduction

In eukaryotes, each nucleosome consists of 147 bp of DNA wrapped twice around a cylindrical protein core containing two copies of each histone: H2A, H2B, H3, and H4 [1,2]. Histone proteins have a structured globular domain and an unstructured amino-terminal tail that protrudes from the core nucleosome. These histone tails can be altered through a variety of post-translational modifications (PTM) including acetylation, phosphorylation, methylation, ubiquitination, and ADP-ribosylation [3]. All these PTMs are reversible, and their dynamics are controlled by two classes of histone-modifying enzymes with opposing effects of addition and removal. Histone PTMs often play an important role in the regulation of various biological processes including transcription, DNA replication and repair, and chromatin dynamics [4,5].

The acetylation state of four core histones is reversible and highly dynamic, with the acetyl group transferred from acetyl-CoA to the N-ε-amino group of lysine residues by histone acetyltransferases (HAT) and removed by histone deacetylases (HDAC) [6]. In general, HATs act as transcriptional activators, since the acetylation of lysine residues in the histone tails can neutralize the positive charge of the nucleosome, thereby disrupting electrostatic interactions between histones and the phosphate groups of DNA, leading to a looser configuration [7,8]. In contrast, HDACs function as transcriptional repressors. Based on the sequence homology and substrate specificity, HDACs are grouped into three major classes in plants: REDUCED POTASSIUM DEPENDENCE 3/HISTONE DEACETYLASE 1 (RPD3/HDA1), SILENT INFORMATION REGULATOR 2 (SIR2) and HISTONE DEACETYLASE 2 (HD2)-related protein families [9]. A large number of studies support the idea that HDACs always associate with other nuclear factors including transcription factors to regulate gene expression [10,11,12]. For instance, HDA6, a member of the RPD3/HDA1 family, is recruited by the transcription factor ARABIDOPSIS PHANTASTICA-LIKE 1 (AS1) to regulate *KNOTTED-LIKE HOMOBOX* (*KNOX*) genes involved in leaf development [13]. HDA6 also associates with FLOWERING LOCUS D (FLD), a lysine-specific demethylase 1 (LSD1)-type histone demethylase, to regulate the expression of *FLC*, *MAF4*, and *MAF5* in flowering control [14]. HDA6 and LSD1-LIKE 1/2 (LDL1/2) form a repressive complex by interacting with CIRCADIAN CLOCK ASSOCIATED 1 (CCA1)/LATE ELONGATED HYPOCOTYL (LHY) and TIMING OF CAB EXPRESSION 1 (TOC1) to repress *TOC1* and *CCA1/LHY* expression, respectively [15,16]. HDA6 also maintains transposable element silencing through directly interacting with DNA METHYLTRANSFERASE 1 (MET1) and the H3K9 methyltransferases SU (VAR) 3-9 HOMOLOG 4/5/6 (SUVH4/5/6) [17,18]. Furthermore, phosphorylation of two serine residues—S427 and S429—of HDA6 results in increased enzymatic activity, whereas a mutation of S427 to alanine in HDA6 abolishes its interaction with SUVH5 and SUVH6, suggesting that the phosphorylation of HDA6 is important for its activity and function [18].

Many studies have demonstrated that the activity of HDACs is regulated by PTMs in mammalian cells [19]. For instance, cigarette smoke extracts (CSE) can induce the SUMOylation of K462 and K51 in HDAC2, and SUMOylated K51 decreases its activity [20]. Indeed, the conjugation of SUMO isoforms (e.g., SUMO1, SUMO2, SUMO3, and SUMO5 in *Arabidopsis thaliana*) to substrates is driven by an E1-E2-E3 enzymatic cascade [21,22]. The SUMO proteins are first acetylated and then bound via a high energy thioester linkage to the heterodimeric SUMO-activating enzyme (E1) [21]. The activated SUMO proteins were then transferred to the SUMO CONJUGATION ENZYME 1 (SCE1) (E2) via transesterification, and were finally donated to substrate proteins by a SUMO-protein ligase (E3). To date, only four *Arabidopsis* SUMO ligases (E3) have been described: SAP AND MIZ1 DOMAINCONTAINING LIGASE1 (SIZ1) [23,24], METHYL METHANESULFONATE-SENSITIVE21 (MMS21 or HIGHPLOIDY2) [25,26], PROTEIN INHIBITORS OF ACTIVATED STATs-LIKE1 (PIAL1), and PROTEIN INHIBITORS OF ACTIVATED STATs-LIKE2 (PIAL2) [27]. Previous studies demonstrated that SUMOylation mediated by SIZ1 has various roles in plant growth [28], secondary cell wall formation [29], flowering [30,31], light response [32,33], immunity [34,35], and metabolism of nutrient elements such as phosphate [23] and nitrogen [36]. Moreover, SIZ1 is implicated in glucose-controlled developmental traits including post-germination growth and root development [37]. Additionally, recent data indicated that SUMOylation mediated by SIZ1 is involved in plant responses to various stresses such as cold [38], heat [39] and drought [40], and is involved in hormone signaling processes such as abscisic acid [41,42], auxin [43], gibberellin [44], and brassinosteroid signaling pathways [45].

In *Arabidopsis*, proteomic data identified a large number of conjugates for SUMO1/SUMO2 mediated by SIZ1 [39,46,47]. Among these, a number of the targets are known to associate with multi-subunit protein complexes including the TOPLESS (TPL) complex, the SWI/SNF chromatin remodeling complex [39], and histone deacetylation-related complexes [39,46]. However, the functions of the proteins SUMOylated by SIZ1 are largely unknown. In the present study, we show that SIZ1 interacts with HDA6 and modulates its function. Overexpression of *HDA6* in the *siz1-3* mutant results in a decreased level of H3 acetylation of the HDA6 target genes *FLC* and *MAF4* compared to the wild-type (WT). Together, these findings indicate that the *Arabidopsis* SUMO E3 ligase SIZ1 interacts with HDA6 and negatively regulates HDA6 function.

## 2. Materials and Methods

### 2.1. Plant Materials and Growth Conditions

All *Arabidopsis* seeds used in this study are in the Columbia background (Col-0). The *hda6* mutant line *axe1-5* [14] and *siz1* mutant *siz1-3* [30] were obtained from the *Arabidopsis* Information Resource Center (http://www.arabidopsis.org/; accessed on 7 July 2014). *proHDA6:HDA6-GFP* transgenic plants were described previously [18]. The full-length cDNA of *HDA6* and *SIZ1* were PCR-amplified and cloned into the pCAMBIA1302 and pHB binary vector, respectively. The *pro35S:HDA6-GFP* (HDA6-OE) and *pro35S:SIZ1-FLAG* transgenic plants were generated using the floral dip method [48]. Double mutants were generated by genetic crossing, and the *axe1-5 siz1-3* double mutant was generated according to Barth’s method [49]. Briefly, crossing homozygous *axe1-5* and *siz1-3* mutants with one another resulted in F_1_ progeny that contained both mutations in the repulsion phase. The outcross progeny, which were generated by crossing the F_1_ plants from the first cross to the male-sterile *ap3-6* mutant [50], were screened by flowering phenotype. The delayed flowering but smaller plants were potentially heterozygous for both mutations. Progeny from these lines were analyzed by PCR for homozygosity of both *axe1-5* and *siz1-3*. F_2_ progeny that were homozygous *axe1-5 siz1-3* and AP3/AP3 were selected for further analysis. All plants were germinated and grown under normal growth light (150–200 μmol photons m^−^^2^ s^−^^1^) at 22 °C under LD (16/8 h light/dark cycle) conditions. A Murashige and Skoog basal salt mixture (MS) with 1.5% sucrose was used as a nutrient source for sample collection.

### 2.2. Gene Expression Analysis

Total RNA was extracted with Trizol reagent (Invitrogen, Carlsbad, CA, USA) according to the manufacturer’s protocol. The first-strand cDNA was synthesized using reverse transcriptase (Takara, Dalian, China). Quantitative PCR (qPCR) analysis was performed using the SYBR Green PCR Supermix (Bio-Rad Laboratories, Hercules, CA, USA) on an ABI7500 Real-Time PCR System (Applied Biosystems, Foster, CA, USA). Each sample was quantified at least in triplicate and normalized using *UBQ10* or *ACTIN2* as an internal control. The gene specific primer pairs for qPCR are listed in Supplementary Table S1. Three biological replicates were performed for qPCR analysis and representative results from one biological replicate are shown.

### 2.3. Protein–Protein Interaction Assay

Yeast two-hybrid assays were performed according to the Matchmaker GAL4-based two-hybrid system 3 protocol (Clontech, San Francisco, CA, USA). The full-length of *HDA6* and *SIZ1* coding sequences (CDS) were subcloned into pGADT7-AD and pGBKT7-BD vectors, respectively. The primers used for the constructs are listed in Supplementary Table S1. The paired AD and BD constructs were co-transformed into yeast strain AH109 using the lithium acetate method [51] and plated on DDO medium (minimal media double dropouts, SD medium lacking tryptophan and leucine) for 3 days at 30 °C. Transformed colonies were plated onto TDO medium (minimal media triple dropouts, SD medium lacking tryptophan, leucine and histidine) containing 40 μg mL^−1^ of 5-bromo-4-chloro-3-indoyl-α-D-galactosidase (TDO/X) to test for possible interactions between HDA6 and SIZ1 under the same conditions.

For BiFC assays, the full-length of both *HDA6* and *SIZ1* CDS was subcloned into the pCR8/GW/TOPO vectors and recombined into YN (pEarleyGate201-YN) and YC (pEarleyGate202-YC) vectors [52], respectively. The YN and YC constructs were used for transient assays by polyethylene glycol (PEG) transfection of *Arabidopsis* protoplasts [53]. Transfected cells were imaged using the TCS SP5 Confocal Spectral Microscope Imaging System (Leica).

Co-immunoprecipitation assay (Co-IP) assays were performed as described previously [54]. *A. tumefaciens* harboring *pEAQ-GFP*, *pEAQ-HDA6-GFP*, *pEAQ-GFP-HDA6,* and *pHB-SIZ1-FLAG* was infiltrated into at least six leaves of tobacco. After 36 h infiltration, tobacco leaves were harvested and ground to a fine powder in liquid nitrogen. Proteins were extracted in an extraction buffer (50 mM Tris-HCl, pH 7.4, 150 mM NaCl, 2 mM MgCl_2_, 1 mM DTT, 20% glycerol, and 1% NP-40) containing protease inhibitor cocktail (Roche, Basel, Switzerland). Cell debris was pelleted by centrifugation at 14,000× *g* for 20 min. The supernatant was incubated with 30 μL of GFP-Trap^®^-A beads (Chromo Tek, Planegg-Martinsried, Germany) at 4 °C for 4 h. Then, the beads were centrifuged and washed six times with a washing buffer (50 mM Tris-HCl, pH 7.4, 150 mM NaCl, 2 mM MgCl_2_, 1 mM DTT, 10% glycerol, and 1% NP-40). Proteins were eluted with 30 μL of 2 × loading buffer and analyzed by Western blotting using anti-GFP (TransGen, HT801-01, Beijing, China) and anti-FLAG antibodies (TransGen, HT201-01, Beijing, China).

### 2.4. Histone Preparations

Histones were isolated from 15-day-old seedlings using sulfuric acid extraction of nuclei followed by acetone precipitation [55]. About 2 g of fresh seedlings were ground in liquid nitrogen, and then 10 mL of NIB buffer (15 mM NaCl, 1 mM CaCl_2_, 60 mM KCl, 5 mM MgCl_2_, 0.7 μg/mL pepstatin, 1 mM phenylmethylsulfonyl fluoride (PMSF), complete mini-Table protease inhibitors (Roche, Basel, Switzerland), 0.8% Triton X-100, and 15 mM PIPES pH 6.8, 0.25 M sucrose) were added. The mixture was filtered through Micra cloth and then centrifuged at 10,000× *g* for 25 min at 4 °C. The nuclei were then extracted twice with 0.4 M H_2_SO_4_ and precipitated with 12 volumes of acetone. The precipitate was collected by centrifugation at 12,000× *g* for 25 min at 4 °C. The pellet was dissolved in 200 μL of 4 M urea.

### 2.5. ChIP Assays

ChIP assays were performed as previously described [56,57]. Chromatin was extracted from 15-day-old seedlings (about 0.3 g) after fixation with formaldehyde, and the chromatin was extracted and then sheared to an average length of 500 bp by sonication. The chromatin was immunoprecipitated with specific antibodies including anti-H3ac (Millipore, 06-599), anti-H3K9ac (Millipore, 07-352, Burlington, MA, USA), and anti-GFP (Abcam, ab290, Cambridge, UK)). An equal amount of the sonicated chromatin solution was set aside as the input sample. After cross-linking was reversed, the amount of precipitated DNA fragments and input DNA was detected by qPCR using specific primers listed in Supplementary Table S1. The relative enrichment of various regions of *FLC* and *MAF4* in mutants over Col-0 was calculated after normalization to *ACTIN2*. The percentage input was calculated by determining 2^−ΔCt^ = 2^−[Ct^_(ChIP)_^−Ct^_(Input)_^]^. The exon region of retrotransposon TA3 [58] was used as a negative control.

### 2.6. SUMOylation Assay

A SUMOylation assay in *Escherichia coli* was performed as previously described [59,60]. The full-length CDS of *HDA6* was cloned into pET28 (a), generating a FLAG C-terminal tag and expressed in the bacteria carrying *pCDFDuet-1-AtSAE1a-AtSAE2* (E1) with *pACYCDuet-1-AtSCE1-AtSUMO1GG* or *pACYCDuet-1-AtSCE1 (C94S)-SUMO1GG* (E2 and SUMO1) [61]. The transformed cells were cultured in LB medium to an OD_600_ of 0.5 and induced by 0.5 mM isopropylthio-β-galactoside. After incubation for 12 h at 25 °C, cells were harvested and used for immunoblotting by anti-FLAG antibody (Sigma, Burlington, MA, USA). MYB30 and HDA19 were used as positive control [46,59].

### 2.7. Statistical Analysis

Data represent the means ± standard error (SE). Differences among treatments were compared by one-way ANOVA followed by a post hoc test with statistical significance set at the level of *p* < 0.05. Statistical analysis was performed using SPSS v16.0 (SPSS Inc., Chicago, IL, USA).

## 3. Results

### 3.1. SUMO E3 Ligase SIZ1 Interacts with HDA6 In Vitro and In Vivo

**Figure 1:**
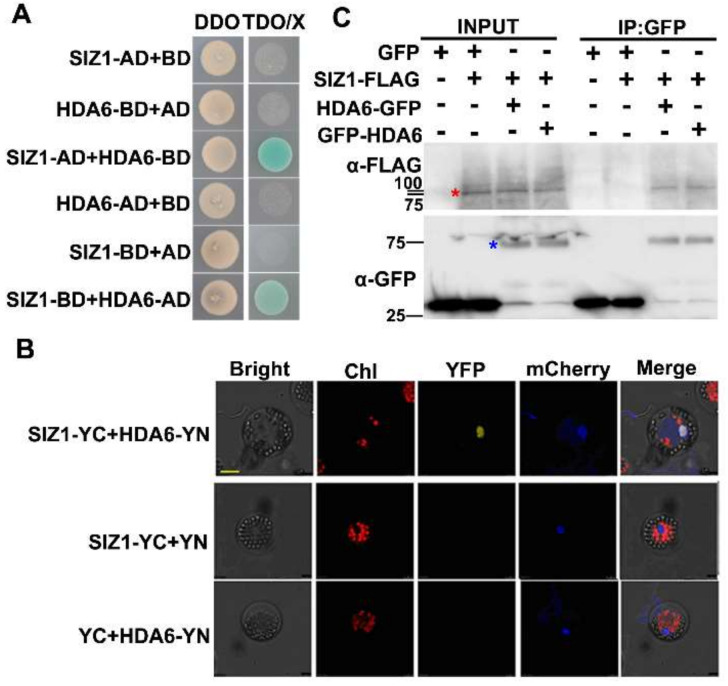
HDA6 interacts with SIZ1 both in vitro and in vivo. (**A**) Yeast two-hybrid analysis of HDA6–SIZ1 interaction. The full-length CDS of *HDA6* and *SIZ1* fused with both the GAL4 activation domain (AD) and GAL4 DNA binding domain (BD) vectors, respectively, and were co-transformed into yeast cells and plated on a DDO medium. The transformants were then plated on TDO/X medium at 30 ℃ to test for a possible interaction. DDO-minimal media double dropouts and the SD medium lacking tryptophan and leucine; TDO-minimal media triple dropouts and SD medium lacking tryptophan, leucine and histidine; TDO/X-TDO medium containing 40 μg mL^−1^ 5-bromo-4-chloro-3-indoyl-α-D-galactosidase. (**B**) BiFC analysis of the interaction between HDA6 and SIZ1 in *Arabidopsis* protoplasts. *SIZ1* and HDA6 were fused with the C-terminus (YC) or the N-terminus (YN) of YFP and co-transformed into *Arabidopsis* protoplasts by PEG-mediated transformation. Empty vectors were used as negative controls. The mCherry was used as a nuclear marker. Chl-auto-fluorescence of chlorophyll. Bars = 25 μm. (**C**) Co-IP analysis of the interaction between HDA6 and SIZ1. The GFP, HDA6-GFP, GFP-HDA6, and SIZ1-FLAG co-expressed in tobacco leaves by *Agrobacterium* injection. Total protein extracts were immunoprecipitated with GFP-Trap-A beads and the immunoprecipitated protein was then detected by Western blotting assays using an anti-FLAG antibody. Input HDA6-GFP and SIZ1-FLAG proteins were detected with anti-GFP and anti-FLAG antibodies, respectively. The molecular weight (kDa) is indicated in the right panel. IP-immunoprecipitation. Red and blue asterisks indicate SIZ1-FLAG and HDA6-GFP (or GFP-HDA6), respectively.

Previous proteomic data demonstrated that HDA19 conjugates with SUMO1/SUMO2 mediated by SIZ1 [46,47]. Since HDA6 is a close homolog of HDA19, we hypothesized that HDA6 may also be a target of SIZ1. Thus, yeast two-hybrid assays were used to detect the interaction between HDA6 and SIZ1. Yeast cells co-transformed with SIZ1-AD (full-length CDS of *SIZ1* fused to pGAKT7) and HDA6-BD (full-length CDS of *HDA6* fused to pGBKT7) were able to grow on the selective medium TDO (minimal media triple dropouts, SD medium lacking tryptophan, leucine and histidine), indicating that SIZ1 could interact with HDA6 in yeast cells (Figure 1A). Similar results were observed when SIZ1 and HDA6 were fused to pGBKT7 and pGAKT7, respectively (Figure 1A).

Next, the interaction between SIZ1 and HDA6 was examined in vivo by bimolecular fluorescence complementation (BiFC) and co-immunoprecipitation (Co-IP) assays. *SIZ1* and *HDA6* were cloned in the YC (pEarleygate-YC) and YN (pEarley gate-YN) vectors, respectively. The YN and YC constructs were then co-delivered into *Arabidopsis* protoplasts. The mCherry carrying a nuclear localization signal was used as a nuclear marker. As shown in Figure 1B, strong YFP signals were observed in the nucleus of *Arabidopsis* protoplasts, suggesting that SIZ1 and HDA6 interact in plant cells. For the Co-IP assays, *HDA6-GFP* or *GFP-HDA6* and *SIZ1-FLAG* constructs were co-expressed into tobacco leaves for analysis. An anti-GFP antibody was used for immunoprecipitation, and the immunoprecipitated proteins were then analyzed by Western blotting assays using an anti-FLAG antibody. Consistent with our previous IP mass data using *proHDA6:HDA6-GFP* transgenic plants [18], the SIZ1-FLAG was co-immunoprecipitated by both HDA6-GFP and GFP-HDA6 (Figure 1C). Similar results were also observed using transgenic plants coexpressing *35S:HDA6-GFP* and *35S:SIZ1-FLAG* (Supplemental Figure S1). These results indicate that SIZ1 interacts with HDA6 both in vitro and in vivo. Notably, two clear bands were detected in the input panel of transgenic plants indicating that SIZ1 may be altered by PTMs in *Arabidopsis*. Indeed, previous proteomic data also demonstrated that SIZ1 can be altered by phosphorylation [62] and SUMOylation [46]. Although the high-molecular-weight form of SIZ1-FLAG was immunoprecipitated by HDA6-GFP, it remains to be determined which PTMs it contains.

Since SIZ1 is a SUMO E3 ligase, we further investigated whether HDA6 can be SUMOylated. First, a rapid method to check the SUMOylation of HDA6 using a reconstituted SUMOylation system with *Arabidopsis* SUMO machinery proteins in *Escherichia coli* was used [59]. Along with a SUMO1 isoform, two types of plasmids were constructed: pACYCDuet, which carried the two protein subunits of the E1 heterodimer, SAE1b and SAE2; and pCDFDuet-1, which carried E2 (SCE1a or an inactive SCE1a-Cys-94-to-Ser (C94S) version of E2 [61]). *HDA6* was cloned into the pET28(a) vector to fuse a FLAG-tag at its C-terminus by PCR. The transcription factors MYB30 and HDA19, two known SUMO target proteins, were used as positive controls [46,59]. The constructed plasmids *pACYCDuet-SAE1b-SAE2*, *pCDFDuet-SUMO1-SCE1a* (or SCE1a (C94S)) and *pET28(a)-HDA6-FLAG* were co-transformed into *Escherichia coli* BL21 (DE3) cells. An anti-FLAG antibody was used to detect the SUMOylation of HDA6. Consistent with previous proteomic data, in vitro SUMOylation reactions resulted in an identical pattern of conjugate bands for HDA19 and MYB30 but not HDA6 (Supplemental Figure S2A). These results indicated that HDA6 may not be a SUMOylation target protein, which is consistent with the predicted results using GPS-SUMO software (http://sumosp.biocuckoo.org/index.php, accessed on 28 October 2021) [63]. Although proteomic data demonstrated that the HDA6 homolog HDA19 is conjugated with SUMO1/SUMO2, the SUMOylation sites of HDA19 were not mapped [46,47]. Nevertheless, it was predicted that the SUMOylation site of HDA19 is located at the C-termini, as HDA6 lacks this motif (Supplemental Figure S2B). Next, *proHDA6:HDA6-GFP* [18] was introduced to *siz1-3* plants by crossing. No difference was found in the level of HDA6-GFP between WT (Col-0) and *siz1-3* (Supplemental Figure S2C), indicating that the expression or stability of HDA6 is not affected by SIZ1.

### 3.2. HDA6 Acts Downstream of SIZ1 to Repress FLC

To explore the function of the interaction between HDA6 and SIZ1, an *axe1-5 siz1-3* double mutant was generated. Since *HDA6* (*AT5G63110*) and *SIZ1* (*AT5G60410*) were located in the same chromosome, we used a crossing scheme to select double mutants, which was used previously to find meiotic recombination between tandemly-duplicated genes *TGG1* and *TGG2* in genus *Arabidopsis* [49]. Briefly, the F_1_ progenies resulting from crossing *HDA6* mutant *axe1-5* [14] and *siz1-3* [30] were chosen as the pollen donors for the second cross to the male-sterile *ap3-6* mutant [50]. F_2_ progenies that were homozygous *axe1-5 siz1-3* were identified by PCR and phenotyping.

**Figure 2:**
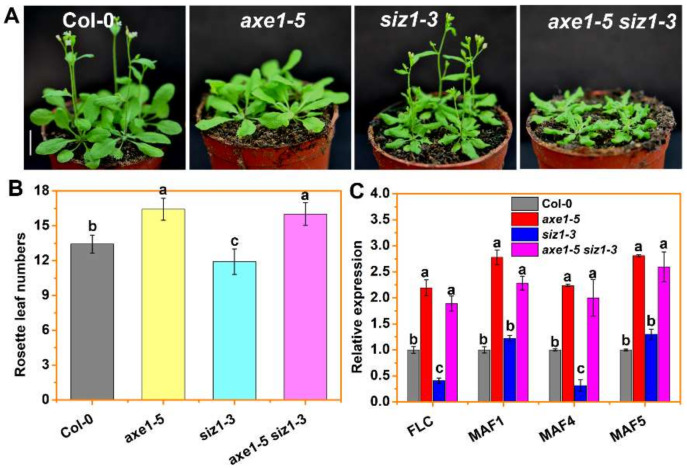
HDA6 acts downstream of SIZ1 to repress *FLC* and *MAF4*. (**A**) Flowering phenotypes of 21-day-old WT(Col-0), *axe1-5*, *siz1-3,* and *axe1-5 siz1-3* plants under LD. Bars = 1 cm. (**B**) Rosette leaf numbers at flowering for WT, *axe1-5, siz1-3,* and *axe1-5 siz1-3* plants under LD. Data are the averages ± SE of three independent replicates and at least 20 plants were scored for each line. Different letters are used to indicate means that are significantly different (*p* < 0.05, post hoc test). (**C**) qRT-PCR analysis of the expression levels of *FLC* and *MAF*s in 15-day-old WT, *axe1-5, siz1-3,* and *axe1-5 siz1-3* plants under LD conditions. *ACTIN2* was used as an internal control. Values are shown as means ± SE. Different letters are used to indicate means that are significantly different (*p* < 0.05, post hoc test). Three independent biological replicates were performed, with similar results obtained.

Consistent with previous studies, *axe1-5* mutant plants exhibited delayed-flowering phenotypes [14], while *siz1-3* showed an earlier-flowering phenotype [30]. The *axe1-5 siz1-3* double mutant plants displayed a late flowering phenotype similar to *axe1-5* under long-day conditions (LD, 16/8 h light/dark) (Figure 2A). Similar results were observed when *axe1-5 siz1-3* plants were grown under short-day conditions (SD, 8/16 light/dark) (Supplemental Figure S3). Next, we also compared the rosette leaf numbers of *axe1-5, siz1-3* and the double mutant *axe1-5 siz1-3*. The flowering time of *axe1-5 siz1-3* and *axe1-5* plants was delayed under LD in terms of the number of rosette leaves at bolting (Figure 2B). In addition, the expression of *FLC* and its clade member *MAF4*, two target genes regulated by HDA6, was significantly upregulated in *axe1-5 siz1-3* and *axe1-5* plants. Meanwhile, the mRNA levels of *MAF1* and *MAF5* were also significantly increased in the *axe1-5 siz1-3* mutant compared to WT and *siz1-3* plants (Figure 2C). Consistent with previous findings [30], the expression of *FLC* and *MAF4* decreased in *siz1-3* plants, which may account for the early flowering phenotype of *siz1-3* plants. Collectively, these results indicate that *HDA6* acts downstream of *SIZ1* in flowering. Previously, we reported that *axe1-5* displayed serrated and twisted leaves under LD conditions [13]. Similar curling and serrated leaves were found in *siz1-3* plants (Supplemental Figure S4). Furthermore, *axe1-5 siz1-3* plants showed more severe curling and serrated leaves compared to the single mutants (Figure 2A, Supplemental Figure S4), suggesting that *HDA6* and *SIZ1* act additively to regulate leaf development.

### 3.3. The H3ac and H3K9ac Levels of MAF4 and FLC Are Decreased in siz1-3 Plants

**Figure 3:**
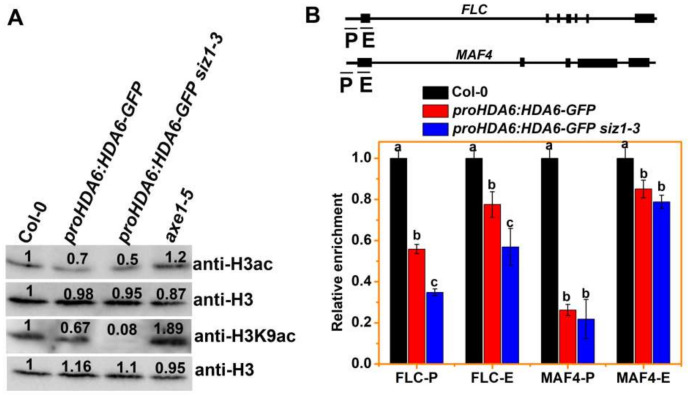
SIZ1 inhibits the activity of HDA6 during flowering. (**A**) Immunoblot analyses of total histone extracted from 15-day-old plants of WT, *proHDA6:HDA6-GFP*, *proHDA6:HDA6-GFP siz1-3*, and *axe1-5* with indicated antibodies. Histone H3 antibody is shown as a loading control. The numbers shown on the gels represent the quantitative results (in arbitrary units). (**B**) Overexpression of *HDA6* decreased the H3ac level on *FLC* and *MAF4* loci. Upper panel: Schematic diagram of *FLC* and *MAF4* loci. P and E represent the promoter and first exon region, respectively. Lower panel: ChIP analysis of H3ac levels on the *FLC* and *MAF4* loci. Fifteen-day-old plant samples were collected for further analysis. The amounts of DNA after ChIP were quantified by qPCR and normalized to *ACTIN2*. Error bars correspond to standard deviations from three biological replicates. Different letters above bars indicate a significant difference between the mutant and WT (*p* < 0.05, post hoc test).

HDA6 is a member of the RPD3/HDA1 group histone deacetylases, which target H3ac and H3K9ac for deacetylation in plants [14,18,64]. Since the SUMO E3 ligase SIZ1 interacts with HDA6 but does not SUMOylate HDA6, we determined whether the deacetylase activity of HDA6 could be inhibited by SIZ1. The deacetylase activity of HDA6 was determined by Western blot (WB) analysis using *proHDA6:HDA6-GFP* transgenic plants, in which *HDA6* was overexpressed (Supplemental Figure S5). The immunoblot analysis indicated that H3ac and H3K9ac levels of *axe1-5* were increased compared to WT (Figure 3A), which is consistent with findings in previous study [14,18,64]. However, the H3ac and H3K9ac levels of *proHDA6:HDA6-GFP siz1-3* plants were decreased compared to WT and *proHDA6:HDA6-GFP* plants (Figure 3A), indicating that the activity of HDA6 was increased with a *siz1-3* background. Moreover, the H3ac levels were decreased in *siz1-3* but increased in *axe1-5 siz1-3* compared to WT (Supplemental Figure S6A). Notably, genome-wide reductions in H3ac and H3K9ac levels did not cause any visible phenotype changes in *proHDA6:HDA6-GFP* compared to WT (Supplemental Figure S7).

Next, we investigated whether overexpressing *HDA6* affects the level of H3ac on its target loci such as *FLC* and *MAF4* [14], by chromatin immunoprecipitation followed by quantitative PCR (ChIP-qPCR). Several HDA6 binding regions of *FLC* and *MAF4*, including their promoters and first exons, were selected for ChIP-qPCR analysis (Figure 3B). We found that the H3ac level of *FLC* and *MAF4* was decreased in *proHDA6:HDA6-GFP* plants in the proximal promoter and first exon regions (Figure 3B), especially in *proHDA6:HDA6-GFP siz1-3* plants compared to WT (Figure 3B). In contrast, the H3ac levels of *FLC* and *MAF4* were increased in *axe1-5* plants in these genomic regions (Supplemental Figure S6B), which is consistent with the increased expression of *FLC* and *MAF4* in *axe1-5* plants. However, the H3ac level of *FLC* and *MAF4* in *axe1-5 siz1-3* was lower than that of *axe1-5*, suggesting that in addition to HDA6, SIZ1 may also regulate the activity of some other histone acetylation enzymes.

### 3.4. SIZ1 Represses HDA6 Binding to Its Target during Flowering

**Figure 4:**
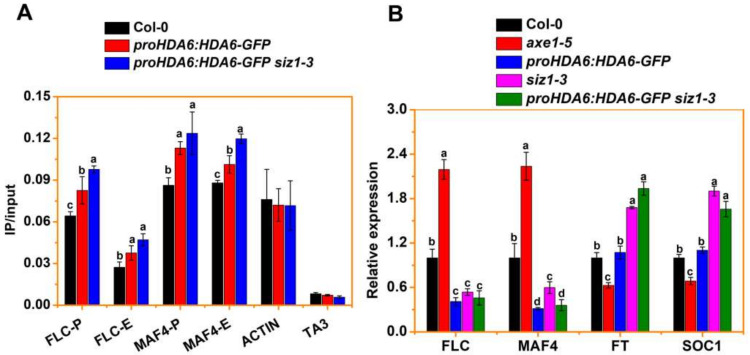
SIZ1 inhibits HDA6 binding to *FLC* and *MAF4* loci. (**A**) ChIP-qPCR analysis of *proHDA6:HDA6-GFP siz1-3* DNA fragments co-immunoprecipitated with the anti-GFP antibody in *FLC* and *MAF4* chromatin. Relative enrichment was calculated based on IP/input for each sample. TA3 and *ACTIN2* were used as the negative control. Values are shown as means ± SE. Error bars correspond to standard deviations from three biological replicates. Different letters are used to indicate means that are significantly different between mutant and WT treatments (*p* < 0.05, post hoc test). (**B**) qRT-PCR analysis of the expression levels of *FLC*, *MAF4*, *FT*, and *SOC1* in 15-day-old WT, *axe1-5*, *proHDA6:HDA6-GFP*, *siz1-3*, and *proHDA6:HDA6-GFP siz1-3* plants under LD conditions. *ACTIN2* was used as an internal control. Values are shown as means ± SE. Error bars correspond to standard deviations from three biological replicates. Different letters are used to indicate means that are significantly different between mutant and WT (*p* < 0.05, post hoc test).

We performed ChIP-qPCR assays using *proHDA6:HDA6-GFP* plants to investigate whether SIZ1 affects the binding profile of HDA6. ChIP-qPCR assays were performed with the anti-GFP antibody and the binding of HDA6 to its target loci, *FLC* and *MAF4,* was analyzed by qPCR. Consistent with previous findings [14], *HDA6-GFP* was significantly enhanced in the first exon and promoter regions of *FLC* and *MAF4* (Figure 3B and Figure 4A). In addition, the binding of HDA6 was analyzed in *proHDA6:HDA6-GFP siz1-3* plants. The binding of HDA6 to the *FLC* and *MAF4* loci was significantly increased in *proHDA6:HDA6-GFP siz1-3* compared to *proHDA6:HDA6-GFP* plants (Figure 4A), suggesting that binding of HDA6 to the *FLC* and *MAF4* loci is repressed by SIZ1. Furthermore, the expression of *FLC* and *MAF4* in both *proHDA6:HDA6-GFP* and *proHDA6:HDA6-GFP siz1-3* plants were significantly reduced compared with WT (Figure 4B). In addition, the expression of *FLOWERING LOCUS T* (*FT*) and *SUPPRESSOR OF OVEREXPRESSION OF CO 1* (*SOC1*), two key genes at the convergence of flowering, was significantly increased in *proHDA6:HDA6-GFP siz1-3* plants compared to WT, but not in *proHDA6:HDA6-GFP* plants (Figure 4B).

## 4. Discussion

Histone deacetylation mediated by HDACs is generally associated with transcriptional repression and gene silencing [10,65,66,67]. By acting as global transcriptional regulators, HDACs always associate with other nuclear proteins (such as transcription factors or chromatin factors) to regulate the expression of genes in plant development and plant response to environmental changes [11,13,14,18,65,66,67]. Furthermore, activities and functions of HDACs are regulated by PTMs, for example, phosphorylation and SUMOylation in mammalian cells [19,68]. In plants, recent studies indicated that phosphorylation of HDA6 can increase its enzymatic activity [18]. Furthermore, proteomic analysis demonstrated that the HDA6 homolog HDA19 is conjugated with SUMO1/SUMO2 mediated by SIZ1 [46,47]. These results indicated that PTMs may also be essential to the functions of HDACs in plants. Our results showed that the SUMO E3 ligase SIZ1 can interact with HDA6 both in vitro and in vivo (Figure 1, Supplemental Figure S1). However, the SUMOylation assays showed that HDA6 is not modified by SUMO1 (Supplemental Figure S2A). SUMOylated proteins typically contain a SUMO modification consensus motif, φKxE, in which φ is an aliphatic residue, preferably L, I or V; K is lysine; X is any residue; and E is glutamate [19]. This motif is found within the C-termini of human HDAC1 and HDAC2 (two SUMOylated proteins) [19]. The SUMOylation sites of HDAC1 were mapped on lysine K444 (VK^444^TE) and K476 (VK^476^EE), and the enzymatic activity and transcriptional repression are affected in the double SUMOylation mutant K444R, K476R (2R) on HDAC1 [69]. HDAC2 has a typical φKxE motif (VK^462^EE) in its unstructured C-terminal domain, which has been identified as a target site for SUMOylation by SUMO1 both in vitro and in vivo [70]. The SUMOylation of HDAC2 is required for NF-κB-dependent gene expression in transformed and primary cells [71]. Notably, although proteomic data demonstrated that the HDA6 homolog HDA19 is conjugated with SUMO1/SUMO2, its SUMOylation sites were not detected [46,47]. Although a predictive consensus motif, VK^482^ME, was found at the C-terminus of HDA19, *Arabidopsis* HDA6 lacks this motif (Supplemental Figure S2B). Furthermore, the expression or stability of HDA6 is not affected by SIZ1 (Supplemental Figure S2B).

In *Arabidopsis*, complex and intricate gene-regulatory networks of transcription regulators guide the flowering time and flower development by integrating both internal and external signals [72]. During flowering, FLC, FLC homologues (such as FLOWERING LOCUS M, MAF2 and MAF4), and the MADS box transcription factor SHORT VEGETATIVE PHASE (SVP), act as negative regulators of flowering time. Generally, high mRNA levels of *FLC* (or *MAFs*) result in later flowering associating with low expression of *FT* and *SOC1*. However, the paradoxical expression pattern of *FLC* (or *MAF4*) and *FT*/*SOC1* was also observed in *brahma* mutants, in which the expression levels of *FLC*, *FT* and *SOC1* were all significantly increased and this was associated with early flowering [73,74]. In accordance with these observations, our results showed that a lower level of H3ac and H3K9ac was associated with reduced expression of *FLC* and *MAF4*, but that this does not lead to earlier flowering in *proHDA6:HDA6-GFP* and *proHDA6:HDA6-GFP siz1-3* plants (Figure 3, Supplemental Figure S7). Consistent with their early flowering phenotypes, the levels of *FT* and *SOC1* transcripts are increased in *proHDA6:HDA6-GFP siz1-3* plants compared to *siz1-3* plants, while the expression of *FT* and *SOC1* is unchanged in *proHDA6:HDA6-GFP* plants compared to WT (Figure 4B). Indeed, FLC, SVP, and MAFs can form several tetrameric repressor complexes with different compositions, such as FLC-SVP-MAF3-MAF4 and SVP-FLM-MAF2-MAF4, to which directly repress the expression of *FT* and *SOC1* [75,76,77]. Moreover, biochemical and genetic results demonstrated that SUMOylation of FLC has a critical role in the regulation of flowering time [31]. Collectively, these results indicate that the modulation of protein levels of SVP and/or FLC clade members that lead to a change in the abundance of a particular complex comprising FLC, MAFs, and/or SVP in response to environmental and endogenous cues, may play a key role in the regulation of flowering time. More experiments are required to explore the roles of HDA6-SIZ1 module in the regulation of *FLC* and *MAFs* in *Arabidopsis*.

Previous studies demonstrated that HDA6 interacts with the histone demethylase FLD and binds to the chromatin of *FLC* and *MAF4* [14]. Meanwhile, SIZ1-mediated SUMO modification of FLD may repress H4 deacetylation of *FLC* chromatin [30]. These findings suggest that HDA6 and FLD may function with SIZ1 to regulate histone deacetylation and demethylation during flowering. Furthermore, genetic analysis indicated that *HDA6* and *FLD* act downstream of *SIZ1* during flowering (Figure 2) [30], suggesting that *SIZ1* is required for the function of *HDA6* and *FLD*. Indeed, overexpression of *HDA6* in *siz1-3* plants caused lower levels of H3ac and H3K9ac compared to WT (Supplemental Figure S5, Figure 3A), indicating that the activity of HDA6 was increased in *siz1-3*. ChIP-qPCR data demonstrated that the binding of HDA6 to the *FLC* and *MAF4* loci was significantly increased in *proHDA6:HDA6-GFP siz1-3* compared to *proHDA6:HDA6-GFP* plants (Figure 4A), suggesting that SIZ1 represses the binding of HDA6 to its target loci. Thus, the lower levels of H3ac and H3K9ac in Col-0 background may be caused by increased binding of HDA6 to chromatin, which is repressed by the interaction with SIZ1. However, since SIZ1 also mediates SUMO modification of other histone acetylation modification enzymes, such as the histone acetyltransferases GCN5 and HAC1, and the histone deacetylase HDA19 [39,46,47], the changes in histone acetylation in *siz1-3* may not be caused by HDA6 alone. Furthermore, a recent study indicated that the PHD finger of *Arabidopsis* SIZ1 recognizes the trimethylated histone H3K4, which mediates the SIZ1 function and abiotic stress response [78]. In human cells, recognition of H3K4me3 by the PHD domains of the ING (for inhibitor of growth) family of tumor suppressor proteins stabilizes the HDAC complex to repress active genes in response to DNA damage [79]. Further research is required to investigate how SIZ1 affects HDAC activity and binding to the target genes.

Similar to *axe1-5* plants, the *axe1-5 siz1-3* double mutants were late flowering under both LD and SD conditions (Figure 2A,B, Supplemental Figure S3), suggesting that *HDA6* acts downstream of *SIZ1* in the floral promotion pathway. However, genetic analysis demonstrated that HDA6 and SIZ1 may act in an additive manner on the pathway to regulate leaf development, since *axe1-5 siz1-3* plants showed more severe curling and serrated leaves compared to WT and the single mutants (Figure 2A, Supplemental Figure S4). A previous study demonstrated that HDA6 is recruited to *KNAT1*, *KNAT2*, and *KNATM* chromatin by the transcription factor AS1, thereby repressing the expression of these genes by downregulating H3ac levels during leaf development [13]. In contrast, SIZ1 plays a role in leaf development by regulating cell division and expansion through SA signaling, which is associated with the expression of *XTH*, encoding xyloglucan endotransglycosylase/hydrolases [80]. Overexpression of a gene encoding bacterial salicylate hydroxylase (*nahG*) in *siz1* plants substantially decreases the levels of SA with normal leaf morphology and rosette plant sizes [30,80]. Collectively, these findings suggest that HDA6 and SIZ1 may have different roles in leaf development. 

In conclusion, our research provides insights regarding the interaction between SIZ1 and HDA6, and their involvement in flowering by regulation of *FLC* and *MAF4* in *Arabidopsis*. The SUMO E3 Ligase SIZ1 represses HDA6 activity and its binding on target genes to induce *FLC* and *MAF4* expression by increasing the levels of histone H3 acetylation.

## Data Availability

All material presented here is available upon request: yangsongguang@scbg.ac.cn and kewu@ntu.edu.tw. The supplementary data are attached at the end of this file.

## Supplementary Materials

The following are available online at https://www.mdpi.com/article/10.3390/cells10113001/s1. Figure S1: Co-IP analysis of the interaction of HDA6 with SIZ1 using transgenic plants. Figure S2: SUMOylation analysis of HDA6 in reconstituted system in *Escherichia coli*. Figure S3: Flowering phenotypes of WT, *axe1-5*, *siz1-3*, *axe1-5 siz1-3* plants grown under SD conditions. Figure S4: Leaf phenotypes of 21-day-old WT, *axe1-5, siz1-3* and *axe1-5 siz1-3* plants under LD. Figure S5: The mRNA levels of *HDA6* in 15-day-old Col-0, *proHDA6:HDA6-GFP* and *proHDA6:HDA6-GFP siz1-3* plants using *ACTIN2* (A) and *UBQ10* (B) as an internal control. Figure S6: The H3ac and H3K9ac levels in *axe1-5, siz1-3, axe1-5 siz1-3* plants. Figure S7: Flowering phenotypes of WT, *axe1-5*, *siz1-3*, *axe1-5 siz1-3*, *proHDA6:HDA6-GFP* and *proHDA6:HDA6-GFP siz1-3* plants under LD. Table S1: Primers used in this study.
