# Supplementary material for: Arabidopsis SUMO E3 Ligase SIZ1 Interacts with HDA6 and Negatively Regulates HDA6 Function during Flowering
Source: Cells. 2021 Nov 3;10(11):3001. doi: 10.3390/cells10113001 (PMC8616286; doi:10.3390/cells10113001)
Supplement: Supplementary file 1 [file cells-10-03001-s001.zip › cells-1334643-supplementary.pdf]

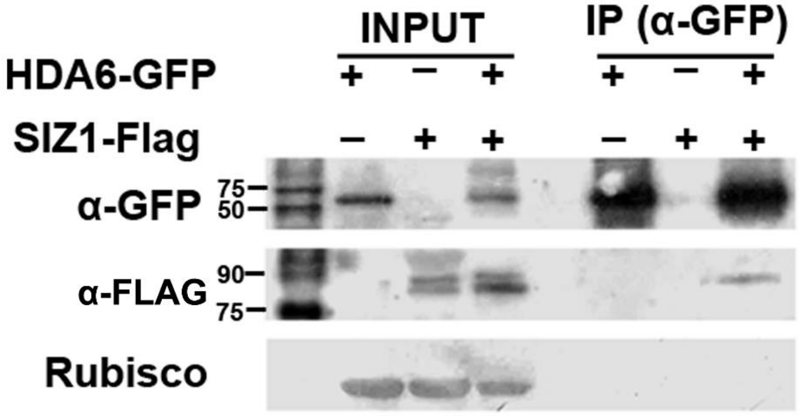

Figure S1. Co-IP analysis of the interaction of HDA6 with SIZ1 using transgenic plants. Total proteins were extracted from 15-day-old co-expressing *pro-HDA6:HDA6-GFP* and *pro35S:SIZ1-FLAG* seedlings grown under LD conditions. GFP-Trap-A beads were used for immunoprecipitation (α-GFP). The precipitated protein and the input samples were detected with anti-GFP and anti-FLAG antibodies. Input HDA6-GFP and SIZ1-FLAG proteins were detected with anti-GFP and anti-FLAG antibodies, respectively. The molecular weight (kDa) is indicated in the right panel. IP-immunoprecipitation, Rubisco indicates loading control.

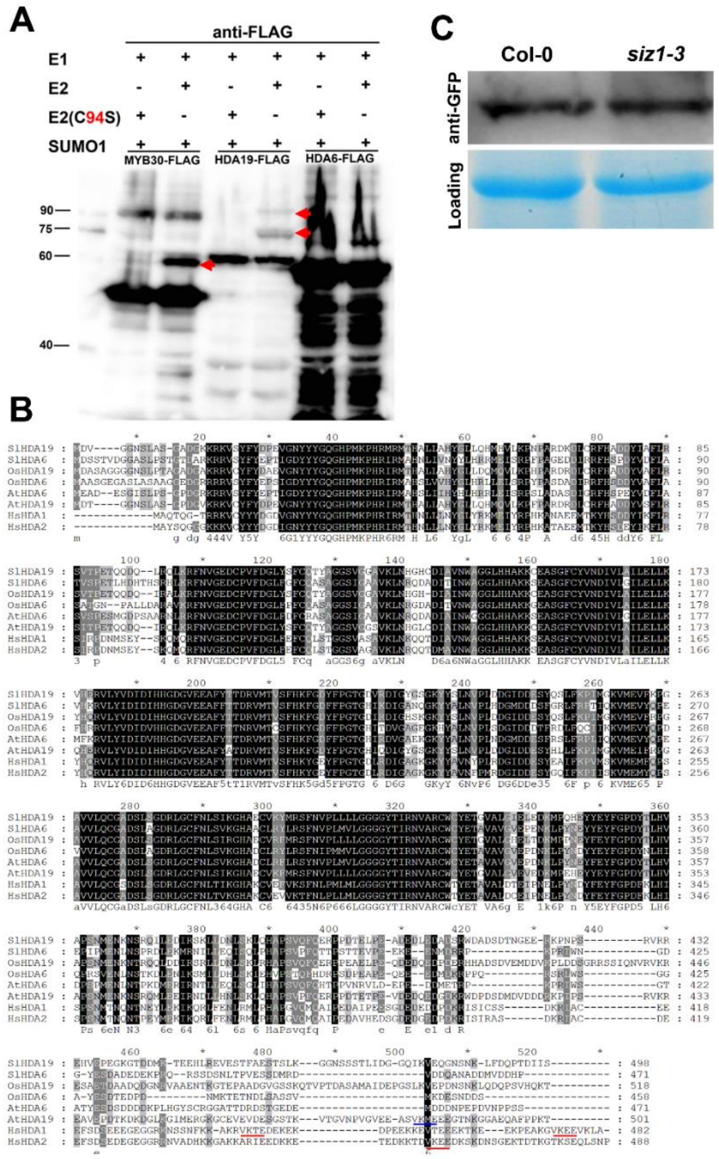

Figure S2. SUMOylation analysis of HDA6. (**A**) The SUMO conjugation of HDA6 was detected in a reconstituted SUMOylation system in *E. coli*. In the presence of E1 and SUMO1 with the E2 or E2 inactive mutant (C94S), the unconjugated and SUMO-conjugated HDA6-FLAGs were detected by an anti-FLAG antibody. MYB30 and HDA19 were used as positive control. The red arrows indicated the SUMO-conjugated of MYB30 and HDA19, respectively. (**B**) The alignment of HDA6 and its homologues in plants and human. At, *Arabidopsis thaliana*; Os, *Oryza sativa*; Sl, *Solanum lycopersicum*; Ho, *Homo sapiens*. The red line indicates the SUMOylation sites of HsHDAC1 and HsHDAC2, while blue line indicates the predictive SUMOylation site of AtHDA19. (**C**) Western blotting analysis of HDA6 protein in *proHDA6:HDA6-GFP siz1-3* transgenic plants. Total proteins were extracted from 15-day-old seedlings in an extraction buffer (50 mM Tris-HCl, pH 7.4, 150 mM NaCl, 2 mM $MgCl_2$, 1 mM DTT, 20% glycerol, and 1% NP-40) containing the protease inhibitor cocktail, subsequently, separated by 10% SDS-PAGE, and detected by western blotting using an anti-GFP antibody. The coomassie blue staining of PAGE gel is shown in the lower panel as a protein loading control.

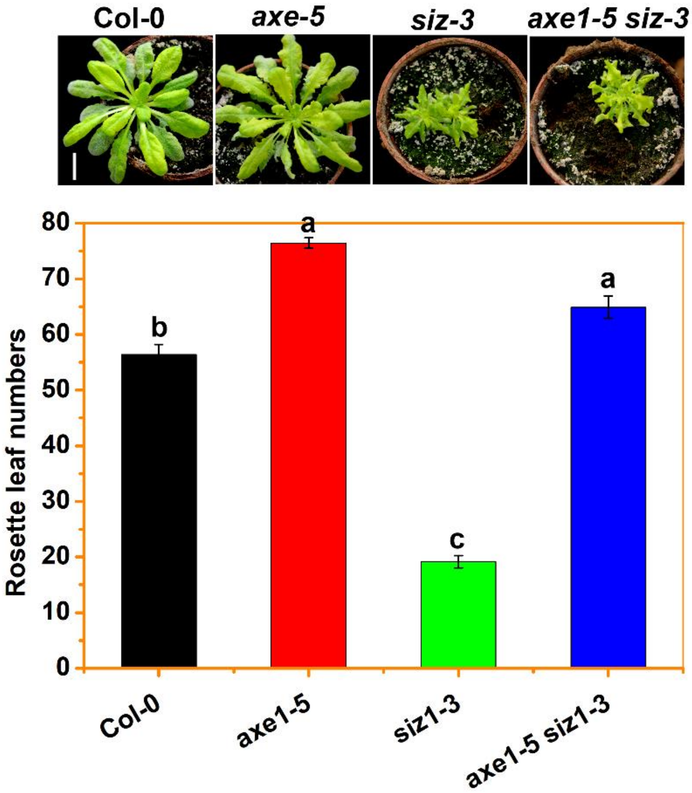

Figure S3. Flowering phenotypes of Col-0, *axe1-5*, *siz1-3*, *axe1-5 siz1-3* plants grown under SD conditions. For direct comparison, the pictures of Col-0, *axe1-5*, *siz1-3*, *axe1-5 siz1-3* grown under SD conditions for 6 weeks were taken at the same time. Bar=1cm.

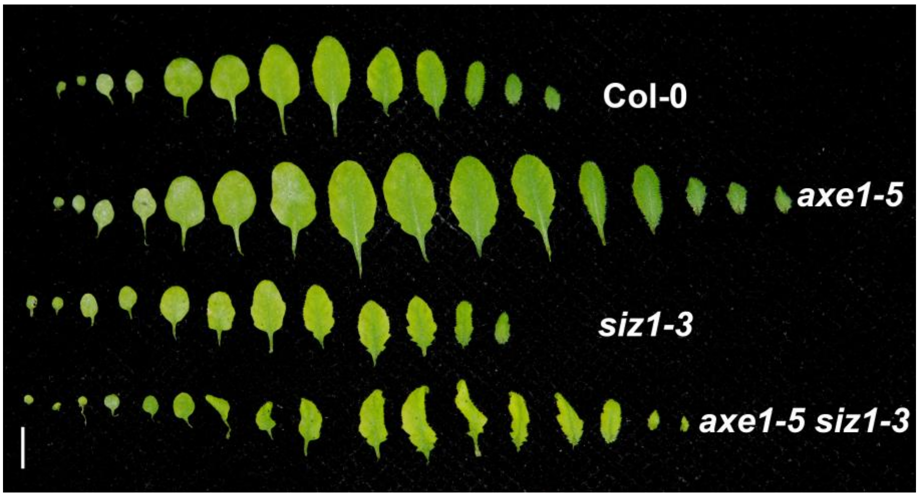

Figure S4. Leaves phenotypes of 21-day-old WT, *axe1-5*, *siz1-3* and *axe1-5 siz1-3* plants under LD. Bars =1 cm.

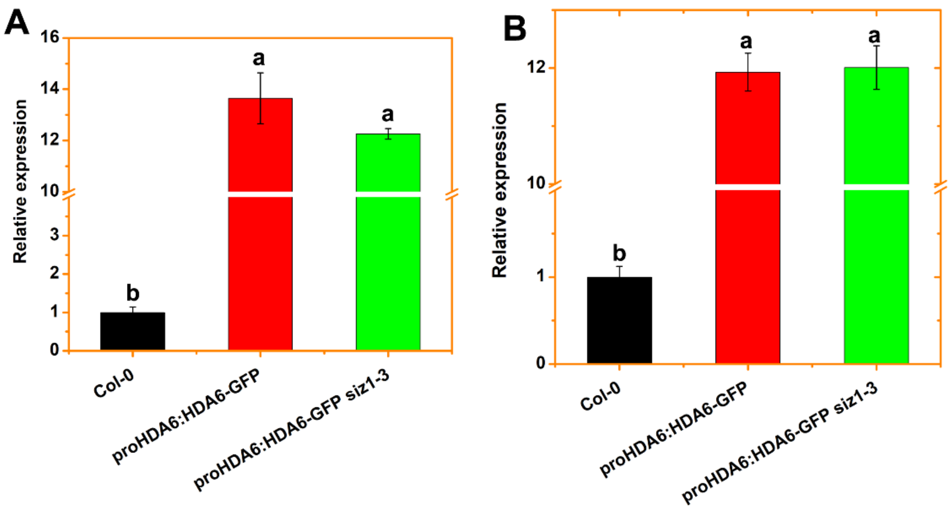

Figure S5. The mRNA levels of *HDA6* in 15-day-old Col-0, *proHDA6:HDA6-GFP* and *proHDA6:HDA6-GFP siz1-3* plants using *ACTIN2* (A) and *UBQ10* (B) as an internal control.

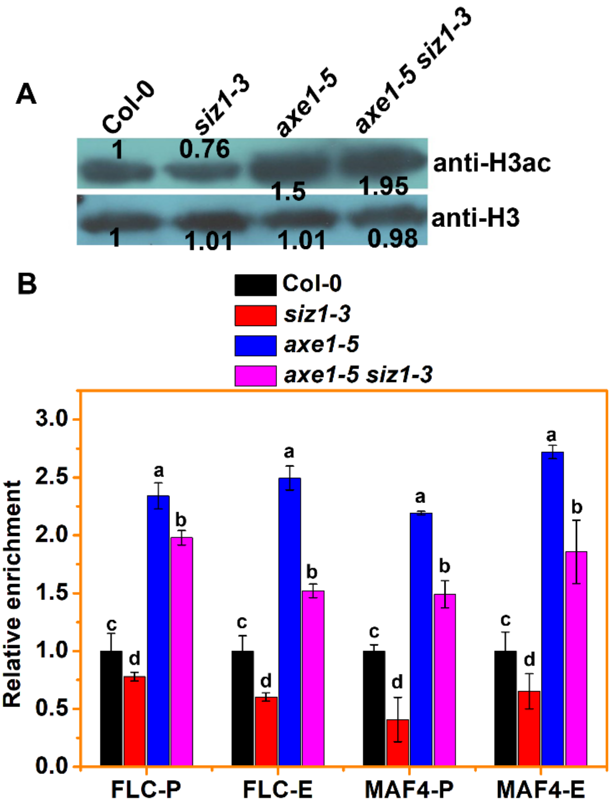

Figure S6. The H3ac levels changes in 15-day-old WT, *axe1-5*, *siz1-3* and *axe1-5 siz1-3* plants. (**A**) Immunoblot analyses of total histone extracted from 15-day-old WT, *axe1-5*, *siz1-3* and *axe1-5 siz1-3* plants using indicated antibodies. The histone H3 antibody was used as a loading control. The numbers shown on the gels represent the quantitative results (in arbitrary units). (**B**) ChIP analysis of H3ac levels on the *FLC* and *MAF4* loci in 15-day-old WT, *axe1-5*, *siz1-3* and *axe1-5 siz1-3* plants. The amounts of DNA after ChIP were quantified by qPCR and normalized to *ACTIN2*. Error bars correspond to standard deviations from three biological replicates. Different letters above bars indicate significant differences between the mutant and WT ($p < 0.05$, post hoc test).

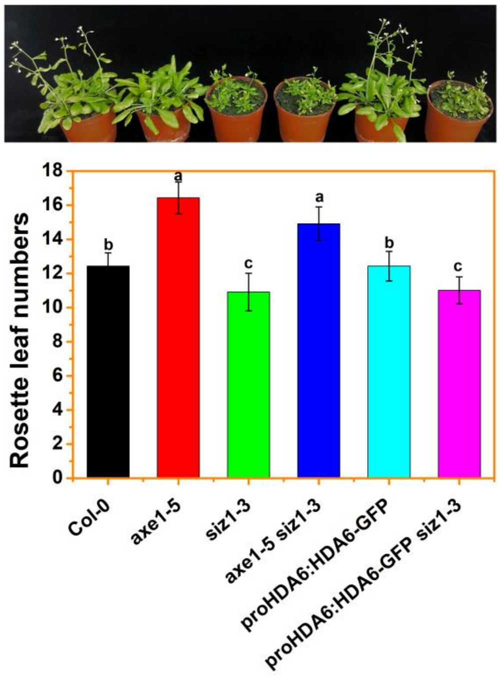

Figure S7. Flowering phenotypes of Col-0, *axe1-5*, *siz1-3*, *axe1-5 siz1-3*, *pro-HDA6:HDA6-GFP* and *proHDA6:HDA6-GFP siz1-3* plants under LD. For direct comparison, pictures of various genetic backgrounds were taken at the same time shortly after bolting of *axe1-5*. Bar=1cm.

Table S1. Primers used in this study.

**Primer for qPCR**	Forward	Reverse
FLC	GCCAAGAAGACCGAACTCAT	TTTGTCCAGCAGGTGACATC
MAF1	TGGTAGTGAGGAAGTGGTTGG	TCGTATGGCCTCTTGTTGTTC
MAF2	GAGACTGCTCTGTCCGTAACTA	GCCAAAGTCTGGTTCTCTTCTC
MAF3	GGGAGAAGTTGCTGATAGAAGAG	AAGAGTCTCCGGTACTTTGTTG
MAF4	ATTAGGTCAGAAGAATTAGTCG-GAGAAAAC	CTTGGATGACTTTTCCGTAGCAGGGGGAAG
MAF5	GGGGATTAGATGTGTCGGAAGAG-TGAAG	GATCCTGTCTTCCAAGGTAACACAAAGG
FT	AG-GCCTTCTCAGGTTCAAAACAAGC	TGCCAAAGGTTGTTCCAGTTGTAGCA
SOC1	ATAGGAACATGCTCAATCGAG-GAGCTG	TTTCTTGAAGAACAAGGTAACCCAATG
UBQ10	GATCTTTGCCGGAAAACAATT-GGAGGATGG	CGACTTGTCATTAGAAAGAAAGAGATAACA
ACTIN2	CTAAGCTCTCAAGATCAAAGGC	AACATTGCAAAGAGTTTCAAGG
**Primer for constructs**		
HDA6-AD	ATGGAGGCCAGTGAATTC<br>ATGGAGGCAG ACGAAAGCGGCA	TCATCTGCAGCTCGAGC<br>AGACGATGGAGGATTCACGTCTG
SIZ1-AD	ATGGAGGCCAGTGAATTC<br>ATGGATTTGGAAGCTAATTG	TCATCTGCAGCTCGAGCGG<br>CTCAGAATCCGAGTCAATGGA
HDA6-BD	CCATGGAGGCCGAATTC<br>ATGGAGGCAG ACGAAAGCGGCA	GCCGCTGCAGGTCGACG<br>AGACGATGGAGGATTCACGTCTG
SIZ1-BD	CCATGGAGGCCGAATTC<br>ATGGATTTGGAAGCTAATTG	GCCGCTGCAGGTCGACG<br>CTCAGAATCCGAGTCAATGGA
SIZ1-pHB-FFLAG	TCTCTCAAGCTTGGATCC<br>ATGGATTTGGAAGCTAATTG	ACCGTCACTAGTGGATCC<br>CTCAGAATCCGAGTCAATGGA
HDA6-pET28-FLAG	GGATCCATGGAT-TACAAGGATGACGACGATAAG<br>ATGGAGGCAGACGAAAGCGG-CATCT	ctcgagtTTAAGACGATGGAGGATTCA<br>CGTCTGGCTCTGGGTTATCGTC
pEAQ-HDA6-GFP	cgCGTCCCGGGTCGAC<br>ATGGAGGCAGACGAAAGCG	TGCTAGTCATTCCTAG<br>AGACGATGGAGGATTCACGT
pEAQ-GFP-HDA6	GGATGAACTATACAAA<br>ATGGAGGCAGACGAAAGCG	GAGTAAAGGCCTCGAG<br>ttaAGACGATGGAGGATTCACGT
HDA6-pCAMBIA-1302	GGACTCTTGACCATGGTA<br>ATGGAGGCAG ACGAAAGCGGCA	GTCAGATCTACCATGGT<br>AGACGATGGAGGATTCACGTCTG
**Primer for ChIP qPCR**		
ACTIN2	CGTTTCGCTTTCCTTAGTGTTAGCT	AGCGAACGGATCTAGAGACTCACCTTG
TA3	GATTCTTACTGTAAAGAACATGG-CATTGAGAGA	TCCAAATTTCCTGAGGTGCTTGTAACC
FLC-P	TGTAGGCACGACTTTGGTAACACC	GCAGAAAGAACCTCCACTCTACATC
FLC-E	CGACTTGAACCCAAACCTGAG-GATCAAAT	AGAAGATAAAAGGGGGAACAAATGAAAAC
MAF4-P	GGTCGGTTTAGAGTCCAATC	TGGTGTAAGATAGTTCCACG
MAF4-E	CGCACCGTTTAGACTCTTTG	GTTGACGAGCTTTCTCCATG
